# Long noncoding RNA *LINC00346* promotes glioma cell migration, invasion and proliferation by up‐regulating ROCK1

**DOI:** 10.1111/jcmm.15899

**Published:** 2020-09-29

**Authors:** Xin Chen, Deheng Li, Lei Chen, Bin Hao, Yang Gao, Liangdong Li, Changshuai Zhou, Xiayun He, Yiqun Cao

**Affiliations:** ^1^ Department of Neurosurgery Fudan University Shanghai Cancer Center Shanghai China; ^2^ Department of Oncology Shanghai Medical College Fudan University Shanghai China; ^3^ Department of Radiation Oncology Fudan University Shanghai Cancer Center Shanghai China

**Keywords:** apoptosis, cell migration, cell proliferation, glioma, *LINC00346*, *miR‐340‐5p*, ROCK1

## Abstract

Long noncoding RNAs have key roles in glioma progression. However, the function and mechanisms of action of the long noncoding RNA, *LINC00346*, in glioma remain unclear. In our study, we observed that *LINC00346* levels were increased in glioma tissue samples, and according to Gene Expression Profiling Interactive Analysis, its levels were related to disease‐free survival and overall survival rates, suggesting that a high level of *LINC00346* expression corresponds to a poor prognosis. We next confirmed the high levels of *LINC00346* expression in glioma tissues and cell lines and showed that *LINC00346* knockdown suppressed glioma cell proliferation, migration and invasion; promoted apoptosis; and delayed tumour growth. Moreover, the oncogenic function of *LINC00346* may be explained, in part, by the down‐regulation of *miR‐340‐5p* and the de‐repression of *ROCK1*. We showed that *LINC00346* may function as a competing endogenous RNA of *miR‐340‐5p*, thereby de‐repressing *ROCK1*. This study revealed a new regulatory network in glioma and identified potential therapeutic targets for this cancer.

## 1 INTRODUCTION

Glioma is the most common, invasive and destructive primary brain tumour, with a poor prognosis and few treatment options.^1^ In the past few decades, progress has been made in surgery, chemotherapy, radiotherapy and combination therapy for the treatment of gliomas, but the median survival time for patients newly diagnosed with glioblastoma remains <15 months.^2^, ^3^ Therefore, it is urgently necessary to identify novel therapeutic targets and gains new insights into the molecular events underlying the pathogenesis of glioma, to enable the development of more effective strategies for its treatment.

Studies have shown that the human genome encodes long noncoding RNAs (lncRNAs, ≥200 nucleotides) that have a limited or no ability to be translated into proteins.^4^, ^5^ Currently, several lncRNAs are known to have vital functions in cellular development, differentiation and various other biological processes.^6^, ^7^, ^8^ Recently, lncRNAs have been shown to perform vital functions in cancer initiation and progression. Many lncRNAs, such as *HAS2‐AS1*, *FOXD2‐AS1*, *LSINCT5* and *SNHG1*, have been shown to promote glioma tumorigenesis.^9^, ^10^, ^11^, ^12^ LncRNA *LINC00346*, which is an intergenic lncRNA, has been found to be involved in many cancers. For example, Shi et al showed that *LINC00346* enhances pancreatic cancer cell proliferation, colony‐forming ability and tumorigenesis.^13^ Xu et al showed that *LINC00346* gene expression levels are increased in gastric cancer, and its expression level positively correlates with a more advanced pathologic stage and poor prognosis.^14^ Yin et al observed that *LINC00346* expression levels are elevated in hepatocellular carcinoma cells, thereby promoting hepatocellular carcinoma progression.^15^ Nonetheless, the function of *LINC00346* in glioma progression remains unclear.

Emerging studies have revealed that lncRNAs and miRNAs are closely related in the regulation of biological processes in cancers, through the competing endogenous RNA (ceRNA) hypothesis.^16^, ^17^ Thus, we speculated that *LINC00346* may function as a ceRNA in glioma. *miR‐340‐5p* has important roles in the progression of many types of human cancers, by functioning as an oncogenic RNA or a tumour suppressor. For example, *miR‐340‐5p* acts as tumour suppressors in non‐small‐cell lung cancer, colon cancer and glioblastoma multiforme (GBM),^18^, ^19^, ^20^ but it acts as an oncogenic RNA in ovarian cancer.^21^ *miR‐340‐5p* expression is down‐regulated in serum exosomes from GBM patients.^22^ Moreover, the *miR‐340‐5p*‐macrophage feedback loop has been shown to modulate tumour progression and the tumour microenvironment in GBM patients and it may represent a prognostic biomarker and a therapeutic target for GBM.^23^ These findings suggest that *miR‐340‐5p* may play a role in glioma progression. Some studies have shown that several lncRNAs, such as *LINC00662*, *LINC01354* and *MCM3AP‐AS1*, may function as *miR‐340‐5p* sponges and thus, affect cancer progression.^24^, ^25^, ^26^ However, the function of these lncRNAs and their mechanisms of action in glioma require further exploration.

In our study, we showed that *LINC00346* levels were increased in glioma tissues and cell lines, and the knockdown of *LINC00346* expression suppressed glioma cell proliferation, migration, and invasion and induced apoptosis in vitro. Moreover, we showed that *LINC00346* may act as a ceRNA of *miR‐340‐5p*, thereby de‐repressing *ROCK1*. These findings will facilitate the development of a novel strategy for the treatment of glioma.

## 2 MATERIALS AND METHODS

### 2.1 Human tissue samples

Twenty glioma tissue samples and 20 normal brain tissue samples were obtained from patients undergoing surgical resection at Fudan University Shanghai Cancer Center. Informed consent was given by all participating patients, and the study protocol was approved by the Research Ethics Committee at Fudan University Shanghai Cancer Center.

### 2.2 Cell culture

Normal human astrocytes (NHAs) and glioma cell lines (U87, U251, LN229 and H4) were obtained from the Shanghai Institutes for Biological Sciences Cell Resource Center. Glioma cell lines were cultured in DMEM (containing 10% foetal bovine serum; Life Technologies, Carlsbad, CA, USA). NHAs were cultured in RPMI 1640 medium (Life Technologies).

### 2.3 Bioinformatics analysis

*LINC00346* expression levels in GBM and normal tissue were analysed using Gene Expression Profiling Interactive Analysis (GEPIA; http://gepia.cancer-pku.cn/).^27^ The prognosis based on *LINC00346* expression levels was also analysed using GEPIA. Potential *LINC00346* and *miR‐340‐5p* binding sites were predicted using miRanda (http://www.miranda.org/) and MiRDB (http://mirdb.org/), and *miR‐340‐5p* and *ROCK1* binding sites were predicted using Targetscan (http://www.targetscan.org/vert_72/).

### 2.4 Cell transfection

Short hairpin RNA (shRNA)‐encoding sequences targeting *LINC00346* were inserted into the pLKO.1 vector at the AgeI and EcoRI sites, to generate *LINC00346* shRNA expression constructs. *LINC00346*‐targeting shRNAs were obtained from Sangon Biotech (Shanghai, China), with the following sequences: 5′‐*CCGG*GCATGAGAACTCTCTGCATCTCGAGATGCAGAGAGTTCTCATGC*TTTTTG*‐3′ and 5′‐*AATTCAAAAA*GCATGAGAACTCTCTGCATCTCGAGATGCAGAGAGTTCTCATGC‐3′. 293T cells were co‐transfected with validated vector and lentivirus packaging vectors (pMD and psPAX2) using Lipofectamine 2000 (Life Technologies). Virus particles were harvested and purified after 48 hours. An *miR‐340‐5p* mimic and inhibitor (anti‐*miR‐340‐5p*) and their relevant negative controls were obtained from RiboBio (*miR10004692‐1‐5* and *miR20004692‐1‐5*; Guangzhou, China).

### 2.5 RNA extraction and quantitative reverse transcription PCR

Total RNA was extracted from tissue samples and cells using TRIzol (Life Technologies). For lncRNA and mRNA analysis, total RNA was reverse‐transcribed into cDNA, and then qPCR was performed on 4 µL of cDNA using a SYBR Green PCR kit (Takara, Dalian, China). lncRNA and mRNA expression levels were normalized to *GAPDH* expression levels. miRNA expression levels were evaluated using Stem‐Loop Primer SYBR Green Quantitative Real‐Time PCR (RiboBio), and the levels were normalized to *U6* expression levels. PCR primer sequences were as follows: *LINC00346* forward, 5′‐CACCATGTTGGCCAGGCTGGT‐3′, reverse, 5′‐GGCCAAAGAGTGACCATCATC‐3′; *miR‐340‐5p* forward, 5′‐CCGTTAGTTACGATTCGAAG‐3′, reverse, 5′‐ AGGCCGCGCGTAGTGATGCAACA‐3′; *GAPDH* forward, 5′‐AGCAAGAGCACAAGAGGAAG‐3′, reverse, 5′‐GGTTGAGCACAGGGTACTTT‐3′; and *U6* forward, 5′‐ AACCTTATATCGGGCGGGA‐3′, reverse, 5′‐TTACGGCGATGCATAAT‐3′.

### 2.6 Cell proliferation

Cell proliferation was assessed using a Cell Counting Kit‐8 (CCK‐8, Beyotime Institute of Biotechnology, Jiangsu, China) assay. Transfected cells were plated in 96‐well plates, and cell viability was evaluated at 24, 48 and 72 hours after seeding. After 2 hours of incubation with the CCK‐8 solution (10 μL), optical density was measured at 450 nm.^28^

### 2.7 Apoptosis quantitation

Flow‐cytometric methods were utilized to analyse apoptosis. After 48 hours of infection, cells were collected, washed and stained with annexin V‐FITC for 15 minutes in the dark at room temperature, according to the manufacturer's instructions. Cells were assessed using flow cytometry (FACScan, BD Biosciences, Franklin Lakes, NJ, USA), and the percentage of stained cells was determined.^29^

### 2.8 Cell migration and invasion assays

The migration and invasiveness of transfected U87 and U251 cells were determined using a QCM Laminin Migration Assay and a Cell Invasion Assay Kit, respectively. After incubation for 48 hours with the migration and invasion assay reagents, absorbance was quantified at 560 nm.

### 2.9 Dual‐luciferase reporter assay

A fragment of the *miR‐340‐5p* gene or the *ROCK1* gene, including the putative *LINC00346‐* or *miR‐340‐5p*‐binding sites, was subcloned into the pmirGLO dual‐luciferase vector, to construct the reporter plasmids, *miR‐340‐5p*‐wild‐type (*miR‐340‐5p*‐wt) or *ROCK1* wild‐type 3′‐untranslated region (UTR, ROCK1‐wt). Mutant putative *LINC00346‐* or *ROCK1*‐binding sites were generated to construct the reporter plasmids, *miR‐340‐5p*‐mutated (*miR‐340‐5p*‐mt) or *ROCK1*‐3′‐UTR‐mutated, (*ROCK1*‐mt). Glioma cells were co‐transfected with the construct(s) and *LINC00346* or *miR‐340‐5p* mimic or negative controls, using Lipofectamine 2000. Luciferase activity was detected using a Dual‐Glo Luciferase Assay System (Promega, Madison, WI, USA), 48 hours after transfection.

### 2.10 RNA immunoprecipitation assay

RNA immunoprecipitation (RIP) assays were performed using the Magna RIP RNA‐Binding Protein Immunoprecipitation Kit (Millipore, Burlington, MA, USA). Briefly, cells were washed and resuspended in ice‐cold RIPA lysis buffer. The resulting cell lysates were centrifuged, and the supernatant was transferred to RIP immunoprecipitation buffer containing AGO2‐ or IgG‐conjugated magnetic beads. After incubation, the magnetic beads were washed and then incubated. RNA was then isolated for quantitative reverse transcription PCR (qRT‐PCR) analysis.^30^

#### 2.10.1 Western blotting

Cells were homogenized and lysed in RIPA buffer including a cocktail of protease inhibitors. Protein extracts were loaded onto 12% polyacrylamide gels, subjected to electrophoresis, and electrotransferred onto nitrocellulose membranes. Protein bands were visualized using an ECL chemiluminescence kit. Antibodies against ROCK1 and β‐actin were obtained from Proteintech (Wuhan, China).

#### 2.10.2 Tumour xenografts

Six‐week‐old male BALB/c nude mice were obtained from Shanghai SIPPR‐BK Laboratory Animal Co., Ltd. (Shanghai, China). Animal experiments were performed in accordance with the guidelines for the care and use of laboratory animals at Fudan University and were approved by the Animal Research Ethics Committee of Fudan University. Transfected sh*LINC00346* or shNC U251 cells (3 × 10^6^ cells) were subcutaneously injected into nude mice. Tumour volume was evaluated every 5 days via the following formula: volume (mm^3^) = length × width^2^/2. Thirty days after injection, the mice were euthanized and the tumours were excised.

#### 2.10.3 Statistical analysis

The experimental results are presented as mean ± SD. Statistical analysis was performed using SPSS 23.0 statistical software (SPSS, Chicago, IL, USA). Student's *t* test and one‐way analysis of variance were used for data analysis. Results with *P* < .05 were considered significant.

## 3 RESULTS

### 3.1 *LINC00346* was up‐regulated in glioma tissues and cell lines

**FIGURE 1:**
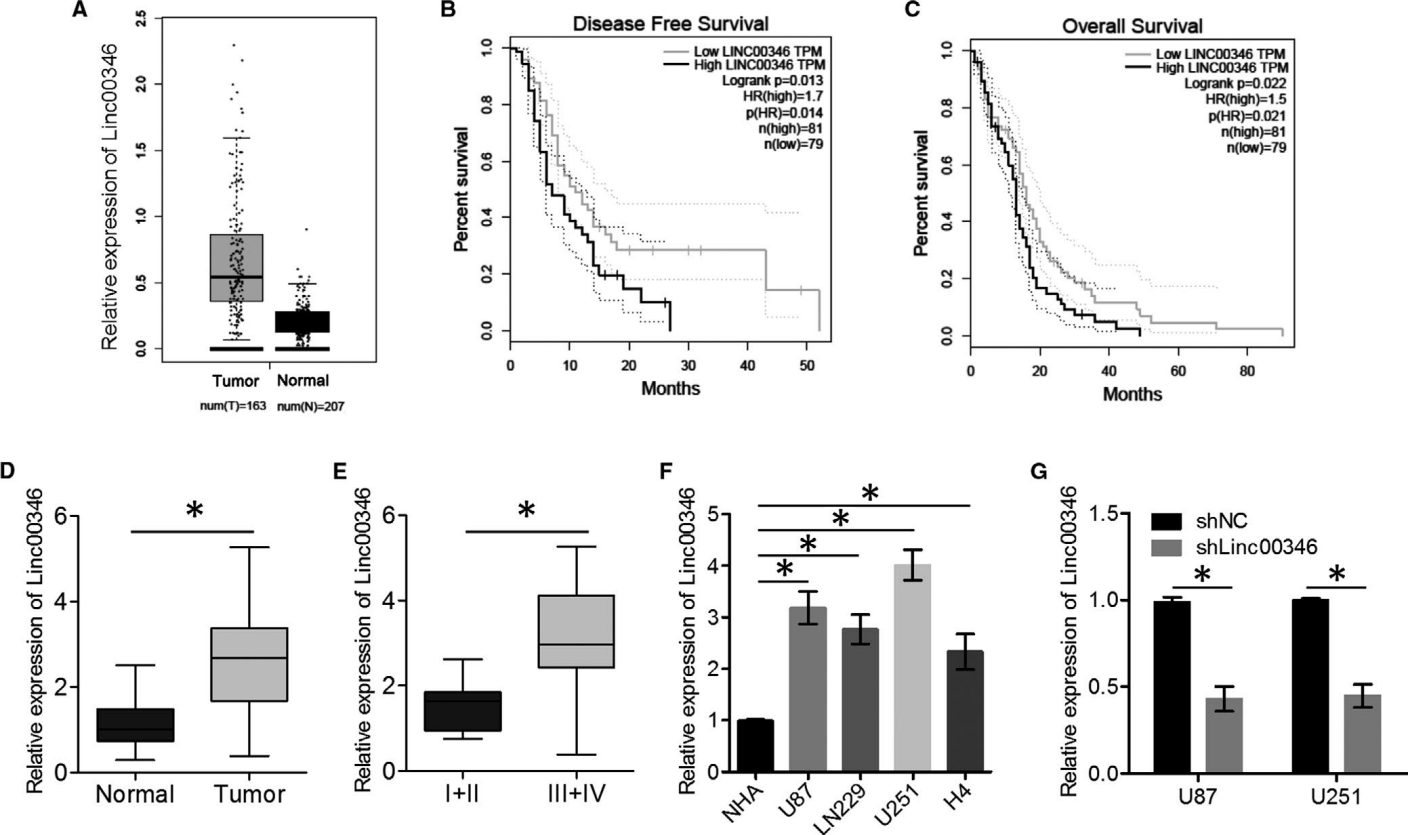
*LINC00346* expression in glioma tissues and cell lines. A, Expression levels of *LINC00346* in GEPIA clinical samples (normal brain tissue and GBM) are presented as a box and whisker plot. B,C, Disease‐free (DFS) and overall (OS) survival rates were computed from TCGA and GTEx data on the GEPIA server. D, The expression levels of *LINC00346* in 20 glioma tissue samples and 20 normal brain tissue samples were measured by qRT‐PCR. E, The expression levels of *LINC00346* in gliomas of different clinical grades. WHO grade I + II, n = 7; II + IV, n = 13. F, The expression levels of *LINC00346* in glioma cell lines and NHAs were determined by qRT‐PCR. U87 and U251 cells were transfected with either sh*LINC00346* or shNC, and *LINC00346* expression was determined by qRT‐PCR. ^*^ *P* < .05

According to the GEPIA database, *LINC00346* expression levels are higher in GBM samples than in their corresponding normal brain tissues (Figure 1A). GEPIA database analysis revealed that *LINC00346* expression was associated with disease‐free and overall survival rates, suggesting that high expression levels of *LINC00346* indicate a poor prognosis (Figure 1B,C). These findings suggested that *LINC00346* may play a role in promoting glioma progression. Our qRT‐PCR results showed that *LINC00346* expression levels were higher in glioma tissues compared with their corresponding normal brain tissues (Figure 1D). Interestingly, *LINC00346* expression was positively correlated with pathological grade (Figure 1E). We also examined *LINC00346* expression in NHAs and a panel of glioma cell lines (LN229, U251, H4 and U87). qRT‐PCR analysis revealed that *LINC00346* levels were clearly increased in glioma cell lines compared with NHAs (Figure 1F). These data suggested that *LINC00346* is up‐regulated in glioma.

### 3.2 *LINC00346* knockdown inhibited the malignant characteristics of glioma in vitro and in vivo

**FIGURE 2:**
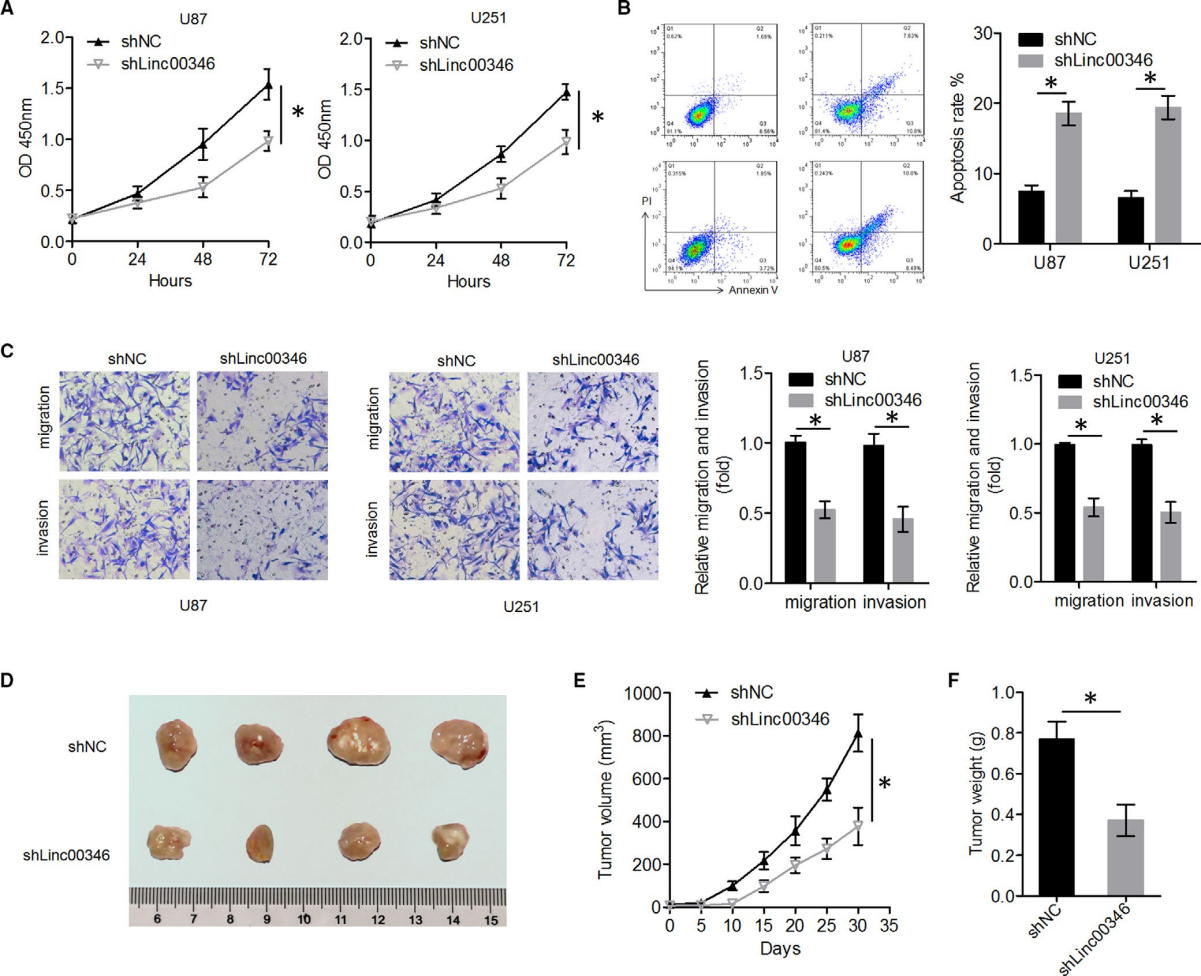
*LINC00346* knockdown suppressed glioma cell proliferation, migration, invasion and tumour growth in vitro and in vivo. A, Cell proliferation was examined using the CCK‐8 assay in U87 and U251 cells after *LINC00346* knockdown. B, The percentage of apoptotic cells was determined by flow cytometry with an annexin V‐FITC antibody and propidium iodide staining. C, Effects of *LINC00346* knockdown on the migration and invasiveness of U87 and U251 cells. D, Xenograft tumours from nude mice. E, The expression levels of *LINC00346* in tumour tissue samples from two groups were measured by qRT‐PCR. F, Tumour growth curves were constructed by measuring tumour volume every 5 d for 30 d after glioma cell injection. G, The weight of tumours excised from the mice in each treatment group was determined on day 30 after injection (n = 4, each group). ^*^ *P* < .05

To explore the biological activity of *LINC00346* in glioma, *LINC00346* expression was stably silenced in U87 and U251 cells (Figure 1F), and the cell proliferation was measured using the CCK‐8 assay, after confirming transfection efficiency. Glioma cells in the sh*LINC00346* group had significantly lower proliferative ability than those in the control group (Figure 2A). The results of flow cytometry showed that the apoptosis rate was higher in the sh*LINC00346* group than in the shNC group (Figure 2B). The migration and invasion abilities of U87 and U251 cells were evaluated using transwell assays. shLINC00346‐transfected cells were found to have significantly lower migration and invasion abilities than shNC‐transfected cells (Figure 2C). Furthermore, the impact of *LINC00346* silencing on tumour growth was determined in vivo. These findings suggested that *LINC00346* knockdown reduced tumour growth, tumour volume and tumour weight (Figure 2D‐G). These data indicated that *LINC00346* knockdown suppressed the malignant characteristics of glioma.

### 3.3 *miR‐340‐5p* knockdown reversed the *LINC00346* knockdown–induced suppression of glioma cell proliferation and invasion

**FIGURE 3:**
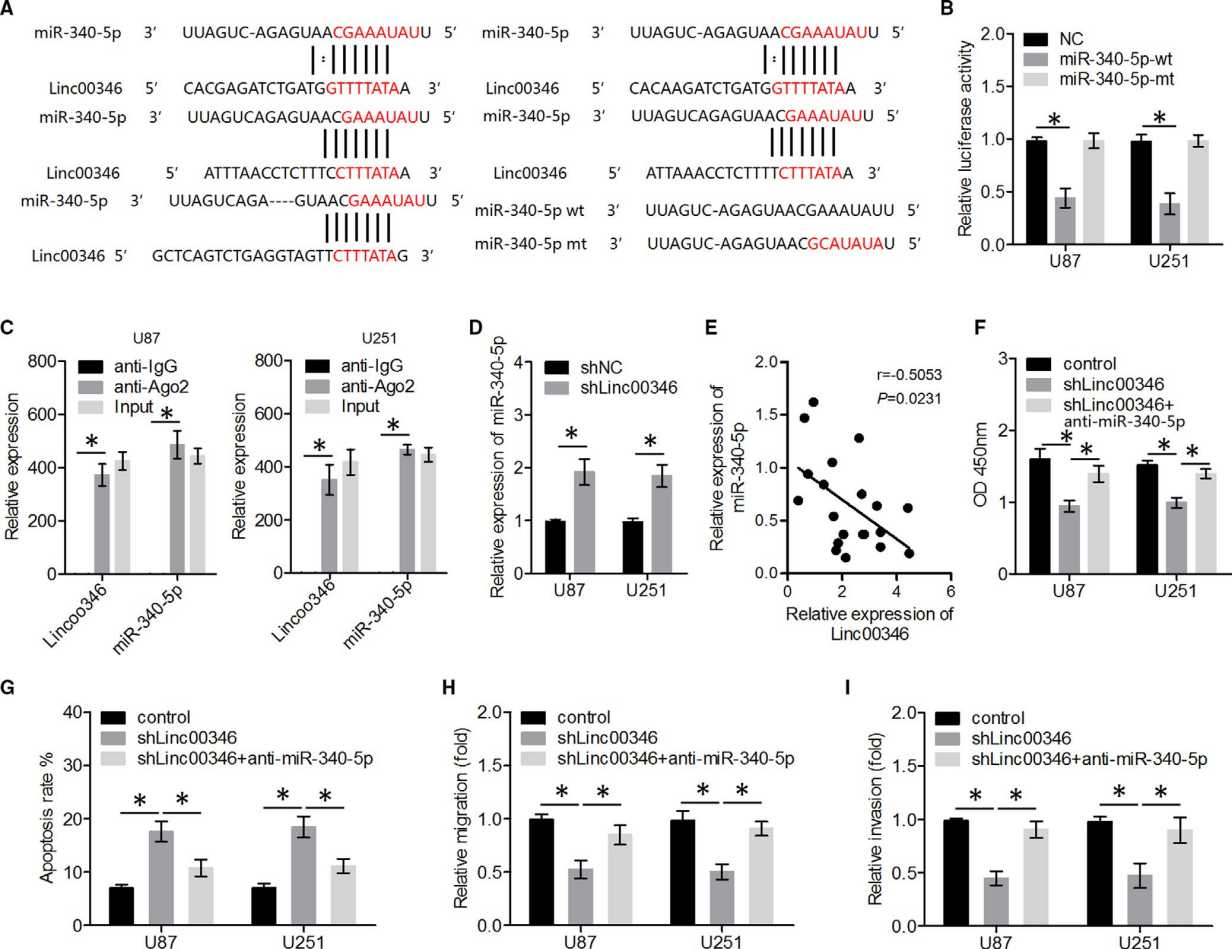
*LINC00346* knockdown significantly inhibited glioma cell proliferation, migration, and invasion and promoted apoptosis by reducing the targeting (sponging) of *miR‐340‐5p*. A, Schematic representation of putative *miR‐340‐5p*–binding sites in *LINC00346*, and the sequence of *miR‐340‐5p*‐mt. B, Dual‐luciferase reporter and (C) RIP assays were performed to determine whether *LINC00346* directly binds to *miR‐340‐5p*. D, The expression levels of *miR‐340‐5p* were assessed in U87 and U251 cells transfected with either sh*LINC00346* or shNC. E, The expression levels of *miR‐340‐5p* were negatively correlated with *LINC00346* expression levels in glioma tissue samples. F‐I, Glioma cell proliferation, apoptosis, and migration and invasion were quantified by CCK‐8, flow cytometry and transwell assays, respectively. ^*^ *P* < .05

Previous studies have shown that lncRNAs function as molecular sponges of miRNAs; thus, we hypothesized that *LINC00346* may have sponging activity. The databases, miRDB and miRanda, revealed that *LINC00346* has five putative binding sites for *miR‐340‐5p*, which serves as a tumour suppressor in several cancers (Figure 3A). Reporter assays showed that the activity of a luciferase gene linked to *LINC00346* was repressed in a dose‐dependent manner in *miR‐340‐5p* mimic‐transfected glioma cells, compared with control cells (Figure 3B). Of note, mutations introduced into the seed sequence of *miR‐340‐5p* (Figure 3A) abrogated its suppressive effects (Figure 3B). Collectively, these data suggested that *LINC00346* directly binds to *miR‐340‐5p* in glioma cell lines.

The RNA‐induced silencing complex is a factor vital for the biological effects of miRNAs, and AGO2 is a key catalytic constituent involved in RNA cleavage.^31^ To determine the possible interactions between *LINC00346* and *miR‐340‐5p*, RIP assays were performed on U87 and U251 cells. As depicted in Figure 3C, the enrichment levels of *LINC00346* and *miR‐340‐5p* were higher in the anti‐AGO2 group than in the anti‐IgG group, suggesting that as a ceRNA, *LINC00346* is capable of sponging *miR‐340‐5p*. We also showed that *LINC00346* knockdown increased *miR‐340‐5p* expression levels in glioma cells (Figure 3D). In addition, we observed that the level of *miR‐340‐5p* expression in glioma tissues was negatively correlated with *LINC00346* levels (Figure 3E). Next, to identify the mechanism by which *LINC00346* regulates the glioma cell activity, U87 and U251 cells were either transfected with sh*LINC0034*6 or co‐transfected with sh*LINC00346* and an *miR‐340‐5p* inhibitor. As presented in Figure 3F‐I, *LINC00346* knockdown suppressed glioma cell proliferation, migration, and invasion and increased apoptosis, whereas *miR‐340‐5p* inhibitor transfection significantly ameliorated the suppressive effects of *LINC00346* knockdown on glioma cells.

### 3.4 *miR‐340‐5p* inhibited glioma cell proliferation, migration and invasion by reducing ROCK1 expression levels

**FIGURE 4:**
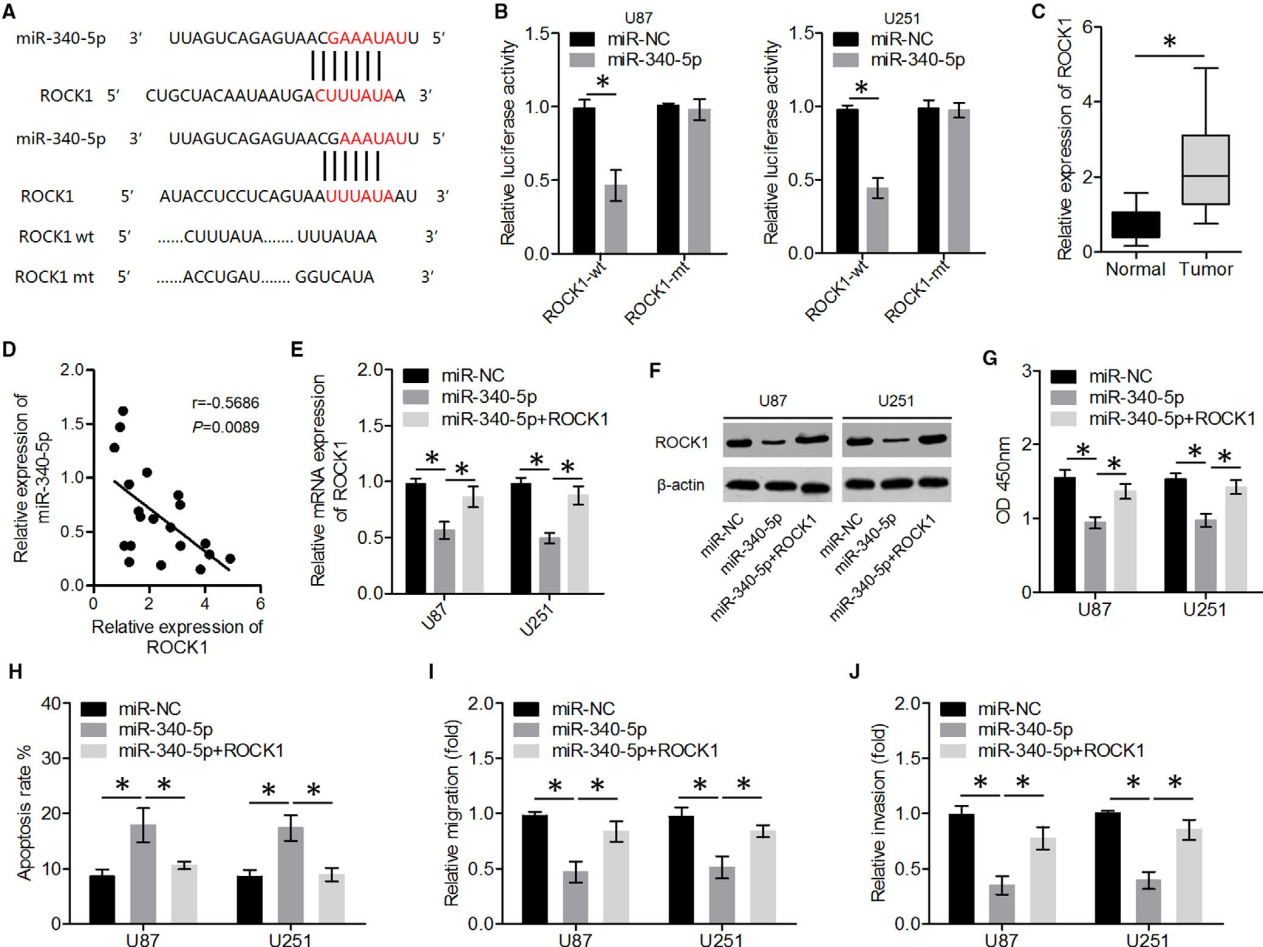
*miR‐340‐5p* inhibited glioma cell proliferation, migration, and invasion and promoted apoptosis by directly targeting *ROCK1* mRNA. A, A schematic representation of putative *miR‐340‐5p*‐binding sites in *ROCK1* mRNA. B, Dual‐luciferase reporter assays were performed to determine whether *miR‐340‐5p* directly binds *ROCK1* mRNA. C, The expression levels of *ROCK1* in glioma tissues and normal brain tissues were quantitated by qRT‐PCR. D, The expression levels of *ROCK1* were negatively correlated with *miR‐340‐5p* expression levels in glioma tissue samples. E, F, The expression levels of ROCK1 were determined in U87 and U251 cells transfected with *miR‐340‐5p* or co‐transfected with *miR‐340‐5p* and a ROCK1‐overexpressing plasmid. G‐J, Glioma cell proliferation, apoptosis, migration and invasion were then measured by CCK‐8, flow cytometry and transwell assays, respectively. ^*^ *P* < .05

We then aimed to identify the target mRNA of *miR‐340‐5p* via bioinformatic analysis. Bioinformatic software predicted that *ROCK1* was the target gene of *miR‐340‐5p* (Figure 4A). To determine whether *ROCK1* was a functional target gene of *miR‐340‐5p*, we constructed the reporter plasmids, *ROCK1*‐wt and *ROCK1*‐mt, which carried the 3′UTR of *ROCK1*, containing either a wild‐type or mutant *miR‐340‐5p*–binding site, respectively. Co‐transfection with an *miR‐340‐5p*‐overexpressing construct decreased the luciferase activity of the *ROCK1*‐wt plasmid but not of the *ROCK1*‐mt plasmid (Figure 4B). In addition, we showed that *ROCK1* levels were higher in glioma tissues than in normal brain tissue, and *ROCK1* levels were significantly negatively correlated with *miR‐340‐5p* levels in glioma samples (Figure 4C,D). Moreover, to clarify whether ROCK1 was involved in the tumour‐suppressive effects of *miR‐340‐5p* in glioma cells, transfection combinations were performed prior to the assessment of glioma cell proliferation, migration, invasion and apoptosis. The results of qRT‐PCR and Western blot assays showed that ROCK1 levels were significantly lower in the *miR‐340‐5p* group, but this difference was smaller in the *miR‐340‐5p* + ROCK1 group (Figure 4E,F).

Overexpression of *miR‐340‐5p* suppressed the proliferation, migration, and invasiveness and promoted the apoptosis of glioma cells, whereas ectopic ROCK1 overexpression significantly attenuated the inhibitory effects of *miR‐340‐5p* on glioma cells (Figure 4G‐I). These results suggested that *ROCK1* is a functional target gene of *miR‐340‐5p* and that ectopic expression of ROCK1 attenuated the tumour‐suppressive effects of *miR‐340‐5p*.

### 3.5 Overexpression of ROCK1 reversed the *LINC00346* knockdown‐induced suppression of glioma cell proliferation and invasion

**FIGURE 5:**
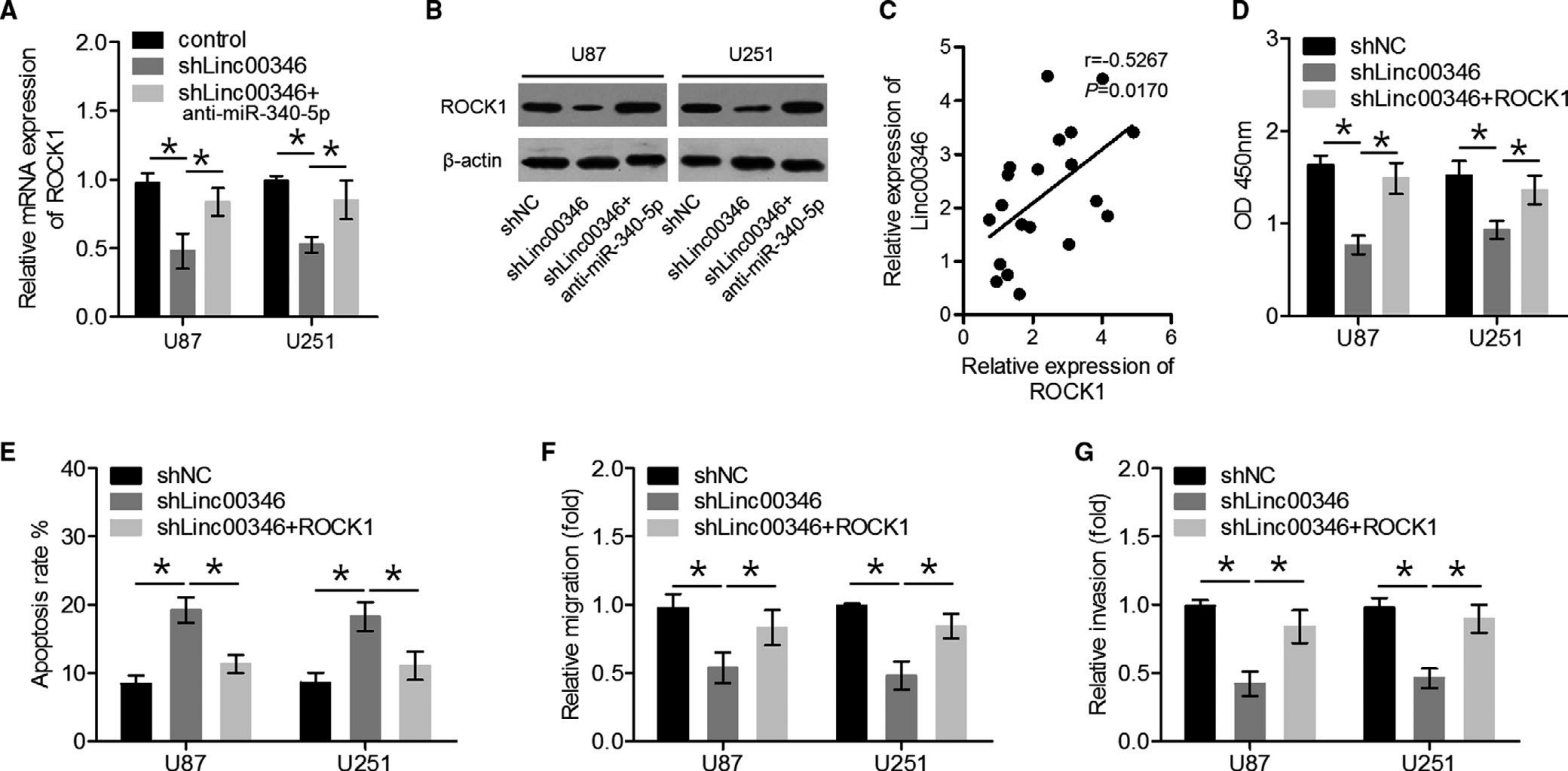
Overexpression of ROCK1 reversed the suppressive effects of *LINC00346* knockdown on glioma cell proliferation and invasion. A, B, The expression levels of ROCK1 were quantified in U87 and U251 cells either transfected with shLINC00346 or co‐transfected with shLINC00346 and an *miR‐340‐5p* inhibitor. C, The expression levels of ROCK1 were positively correlated with *LINC00346* expression levels in glioma tissue samples. D‐G, Glioma cell proliferation, apoptosis, migration and invasion were quantitated by CCK‐8, flow cytometry and transwell assays, respectively. ^*^ *P* < .05

Transfection of an *miR‐340‐5p* inhibitor clearly restored ROCK1 levels in sh*LINC00346*‐transfected cells (Figure 5A,B). In addition, we showed that ROCK1 levels positively correlated with *LINC00346* levels in glioma tissues (Figure 5C). We performed rescue experiments to confirm that ROCK1 mediated the effects of *LINC00346* on glioma cell growth and invasion. As expected, overexpression of ROCK1 reversed the suppressive effects of *LINC00346* knockdown on glioma cell proliferation, migration, and invasion and the stimulatory effect on glioma cell apoptosis (Figure 5D‐G). Overall, these data revealed that the knockdown of *LINC00346* suppressed glioma progression, because of ROCK1 down‐regulation. Therefore, *LINC00346* may function as a ceRNA of *miR‐340‐5p*, thus up‐regulating ROCK1.

## 4 DISCUSSION

Glioma is one of the most common malignant brain tumours; therefore, investigation of the molecular mechanisms involved in glioma progression is essential for the effective treatment of this cancer.^32^ *LINC00346* levels are increased in hepatocellular carcinoma, pancreatic cancer and gastric cancer, thus contributing to cancer progression.^14^, ^15^, ^33^ In the present study, GEPIA data analysis revealed that *LINC00346* was up‐regulated in GBM tissue samples and was associated with a poor prognosis, suggesting that *LINC00346* may be involved in glioma progression. In addition, we confirmed higher *LINC00346* expression levels in glioma cell lines and glioma samples than in normal cells and tissues. Moreover, we observed that the knockdown of *LINC00346* suppressed glioma cell proliferation, migration, and invasion and promoted apoptosis in vitro, and inhibited tumour growth in vivo. These findings revealed that *LINC00346* serves as an oncogenic RNA in glioma and contributes to the progression of this cancer.

Mounting evidence points to a close link between lncRNAs and miRNAs in the regulation of various biological processes.^16^ We predicted *miR‐340‐5p* to be the target miRNA of *LINC00346* using the bioinformatic tools, miRDB and miRanda. This finding was confirmed using dual‐luciferase reporter, qRT‐PCR and RIP assays. *miR‐340‐5p* performs an antitumour function in lung cancer, pancreatic cancer, ovarian cancer and angiosarcoma.^34^, ^35^, ^36^, ^37^ Li et al reported that *miR‐340‐5p* suppresses glioblastoma cell proliferation by repressing CDK6, cyclin D1 and cyclin D2.^38^ The *miR‐340‐5p*‐macrophage feedback loop modulates the progression of GBM and may represent a prognostic biomarker and a therapeutic target for GBM [23]. In our study, we showed that *miR‐340‐5p* interacted with *LINC00346*, and that *miR‐340‐5p* levels negatively correlate with *LINC00346* levels in glioma tissues. Furthermore, an *miR‐340‐5p* inhibitor attenuated the antitumour effects of *LINC00346* knockdown in glioma cells. These results suggested that *LINC00346* knockdown inhibited glioma progression via up‐regulation of *miR‐340‐5p*.

Bioinformatic software predicted that binding sites for *miR‐340‐5p* are present in *ROCK1* mRNA. Next, we found that *ROCK1* was a downstream target of *miR‐340‐5p*, which is consistent with data from a previous study.^39^ ROCK1 is a member of the Rho‐associated protein kinase family and has a role in cell invasion in neoplasms.^40^ ROCK1 is also overexpressed in several cancers, such as hepatocellular carcinoma, non‐small cell lung cancer, breast cancer and oral squamous cell carcinoma.^41^, ^42^, ^43^, ^44^ An et al showed that *miR‐124* suppresses glioma cell invasion by down‐regulating ROCK1.^45^ In addition, ROCK1 has been shown to be a novel target of *miR‐145*, thus promoting glioma cell invasion.^46^ An miRNA can target several mRNAs and an mRNA can also be regulated by one or several miRNAs involved in several different processes. *LINC00346* may also be able to regulate the expression of ROCK1 through *miR‐145* or *miR‐124*, thus participating in the development of glioma. This study focused on *miR‐340‐5p*. The roles of *miR‐145* and *miR‐124* will be investigated in future studies. Here, we found that ROCK1 levels were negatively correlated with *miR‐340‐5p* levels, but were positively correlated with *LINC00346* levels in glioma tissues. We also showed that an *miR‐340‐5p* mimic suppressed the proliferation, migration, and invasiveness and promoted the apoptosis of glioma cells, whereas ROCK1 overexpression abrogated these effects of *miR‐340‐5p* overexpression. Furthermore, we demonstrated that *LINC00346* promotes glioma cell proliferation, migration, and invasion and inhibits apoptosis by increasing the output of the *miR‐340‐5p*‐ROCK1 axis.

In conclusion, we found that *LINC00346* levels were increased in glioma tissues and cell lines and *LINC00346* knockdown suppressed glioma cell proliferation, migration, and invasion and promoted apoptosis in vitro, and inhibited tumour growth in vivo. Moreover, the oncogenic role of *LINC00346* in glioma may be partly explained by the inactivation of *miR‐340‐5p* and thus, the disinhibition of ROCK1 expression. This study uncovered a new regulatory network in glioma and may help identify potential therapeutic targets for glioma.

## CONFLICT OF INTEREST

The authors confirm that there are no conflicts of interest.

## AUTHOR CONTRIBUTION

**Xin Chen:** Investigation (lead); Writing‐original draft (lead). **Deheng Li:** Investigation (equal). **Lei Chen:** Investigation (lead); Resources (equal); Writing‐original draft (lead). **Bin Hao:** Data curation (equal); Software (equal). **Yang Gao:** Formal analysis (equal); Methodology (equal); Validation (equal). **Liangdong Li:** Methodology (equal); Resources (equal); Validation (equal). **Changshuai Zhou:** Investigation (equal); Supervision (equal); Visualization (equal). **Xiayun He:** Funding acquisition (supporting); Project administration (lead); Writing‐review & editing (lead). **Yiqun Cao:** Formal analysis (equal); Funding acquisition (supporting); Project administration (lead); Writing‐review & editing (lead).
